# 2733. Evusheld (AZD7442) Uptake and COVID-19 Outcomes in Solid Organ Transplant Recipients: A Secondary Analysis

**DOI:** 10.1093/ofid/ofad500.2344

**Published:** 2023-11-27

**Authors:** Haya Hayek, Tess Stopczynski, Asim Khanfar, Yasmeen Z Qwaider, Justin Z Amarin, Madge I Stuhlreyer, Samar Alsabah, Samantha Economos Frank, Mackenzie Johnson, Roman Perri, Kelly Schlendorf, Kelly A Birdwell, Kevin C Dee, Daniel Dulek, Andrew J Spieker, James Chappell, Natasha B Halasa

**Affiliations:** Vanderbilt University Medical Center, Nashville, Tennessee; Vanderbilt University Medical Center, Nashville, Tennessee; Vanderbilt University Medical Center, Nashville, Tennessee; Vanderbilt University Medical Center, Nashville, Tennessee; Vanderbilt University Medical Center, Nashville, Tennessee; Vanderbilt University Medical Center, Nashville, Tennessee; VUMC, Nashville, Tennessee; Vanderbilt University Medical Center, Nashville, Tennessee; Vanderbilt University Medical center, Franklin, Tennessee; Vanderbilt University Medical Center, Nashville, Tennessee; Vanderbilt University Medical Center, Nashville, Tennessee; Vanderbilt University Medical Center, Nashville, Tennessee; Vanderbilt University Medical Center, Nashville, Tennessee; Vanderbilt University Medical Center, Nashville, Tennessee; Vanderbilt University Medical Center, Nashville, Tennessee; Vanderbilt University Medical Center, Nashville, Tennessee; Vanderbilt University Medical Center, Nashville, Tennessee

## Abstract

**Background:**

Evusheld is a pre-exposure prophylaxis for COVID-19. It was recommended for high-risk populations, including solid organ transplant (SOT) recipients, due to their increased risk of severe COVID-19 and suboptimal vaccine response. Evusheld received emergency use authorization (EUA) in 12/2021, which was withdrawn on 1/27/2023 due to limited strain efficacy. This study examines Evusheld uptake among SOT recipients and evaluates the frequency and outcomes of COVID-19 infections in those receiving at least one dose.

**Methods:**

We performed a secondary analysis using data from adult SOT recipients (heart, kidney, liver) enrolled in a phase II, multicenter, randomized controlled, double-blind immunogenicity and safety clinical trial for influenza vaccines. Our study included patients enrolled at Vanderbilt University Medical Center between 1/11/2021 and 2/28/2023 who had undergone transplantation before 1/27/2023. Demographic characteristics, transplant history, and COVID-19 vaccine information were obtained from the study database. We conducted chart abstractions until 4/27/2023 to collect data on COVID-19 infections, outcomes, and Evusheld receipt and dosage.

**Results:**

Our study included 62 heart, 93 kidney, 14 liver, and 12 multiple-organ transplant recipients, for a total sample size of 181. Overall, 99 patients (54.7%) received Evusheld. We did not find sufficient evidence of differences in demographic characteristics or frequency of symptomatic COVID-19 infections between those who received Evusheld vs. those who did not. None of the 14 liver transplant recipients received Evusheld, and patients who received Evusheld were more likely to have been transplanted during the EUA period (**Table 1**). The number of posttransplant COVID-19 infections was 0.09 per person-years in those who did not receive Evusheld. Among those who received Evusheld, the number of posttransplant COVID-19 infections was 0.14 per person-years pre-Evusheld and 0.08 post-Evusheld (**Table 2**; **Figure**).

**Figure:**
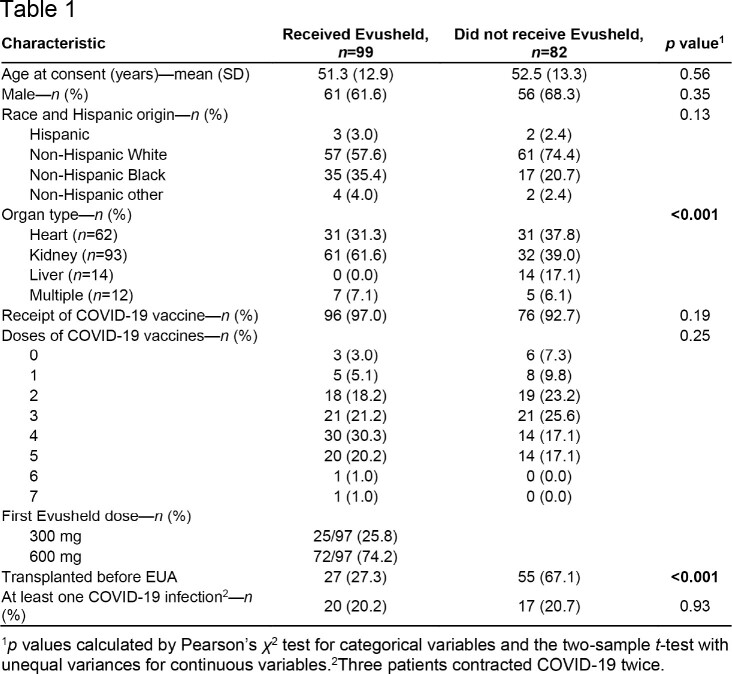

Demographics, clinical characteristics, and COVID-19 outcomes of adult solid organ transplant recipients who received a transplant between 08/09/2020 and 01/22/2023, stratified by Evusheld receipt (N=181).

**Figure:**
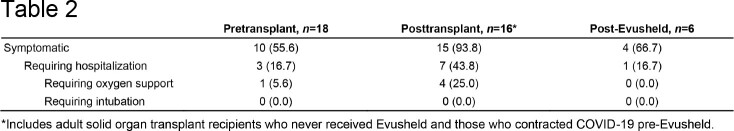

Timing and characteristics of COVID-19 infections in 181 adult solid organ transplant recipients.

Figure

**Figure:**
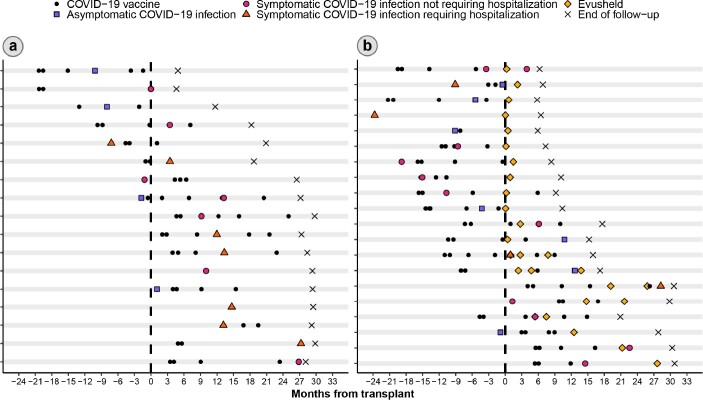

Timeline of COVID-19–related events in those who tested positive for COVID-19 at least once and (a) did not receive Evusheld or (b) received at least one dose of Evusheld.

**Conclusion:**

Despite recommendations, Evusheld uptake among SOT recipients in our cohort was suboptimal. Additional research is needed to understand factors associated with suboptimal Evusheld uptake and to evaluate the effectiveness of monoclonal antibodies against COVID-19.

**Disclosures:**

**Daniel Dulek, MD**, Eurofins Viracor: Research supplies **Natasha B. Halasa, MD, MPH**, Merck: Grant/Research Support|Quidell: Grant/Research Support|Quidell: donation of kits|Sanofi: Grant/Research Support|Sanofi: vaccine support

